# Integrator complex subunit 6 (INTS-6) mediates DNA damage response in *Caenorhabditis elegans*

**DOI:** 10.17912/micropub.biology.001345

**Published:** 2024-11-06

**Authors:** Cristina Romero-Aranda, Beatriz Sáenz-Narciso, Eva Gómez-Orte, Ángela Metola, Begoña Ezcurra, Olga Calvo, Hilde Nilsen, Antonio Miranda-Vizuete, Juan Cabello

**Affiliations:** 1 Center for Biomedical Research of La Rioja (CIBIR), Logroño, La Rioja, Spain; 2 Instituto de Biologia Funcional y Genomica (IBFG), CSIC-Universidad de Salamanca, Salamanca, Spain; 3 Department of Microbiology, Oslo University Hospital PO Box 0424 Oslo, Norway. University of Oslo, The Norwegian Centre on Healthy Ageing (NO-Age), Oslo, Norway. CRESCO- Centre for embryology and healthy development, University of Oslo, Norway.; 4 Instituto de Biomedicina de Sevilla, IBIS/Hospital Universitario Virgen del Rocío/CSIC/Universidad de Sevilla, Sevilla, Spain

## Abstract

The *Caenorhabditis elegans* Integrator complex is a set of at least 13 evolutionarily conserved proteins that binds the C-terminal domain of RNA polymerase II to regulate snRNA 3'-end processing and gene expression. Here we show that the Integrator subunit 6 intervenes in the DNA damage response in *C. elegans* . We find that upon X-ray radiation, INTS-6 is necessary for RAD-51 foci formation. In addition, CDK-1 Tyr-15 phosphorylation depends on the presence of INTS-6 . This work adds a new piece to elucidate the Integrator complex mechanism of action in DNA repair.

**Figure 1. Integrator complex subunit 6 (INTS-6) mediates DNA damage response in Caenorhabditis elegans:**
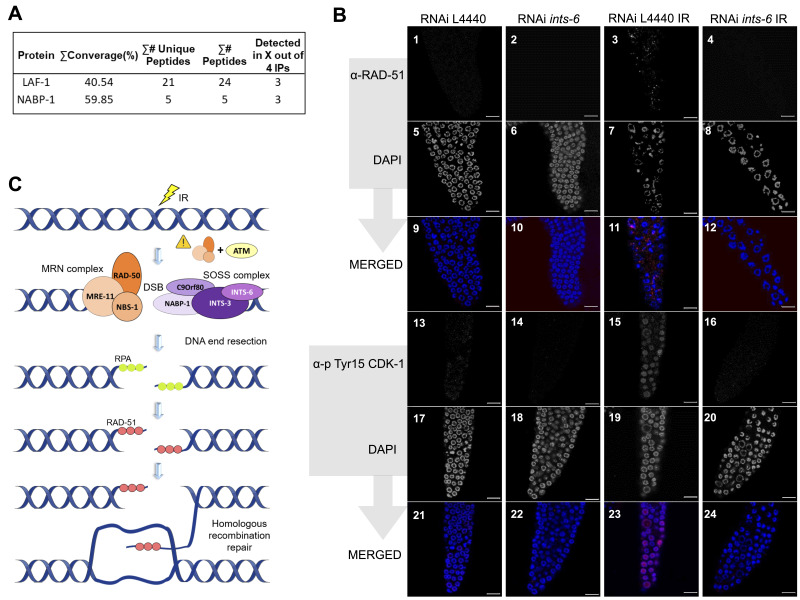
**(A)** INTS-6-associated proteins identified by anti-FLAG affinity purification. In addition to members of the Integrator complex, other proteins were immunoprecipitated along with INTS-6::3xFLAG::GFP, including some related to DNA damage response, such as LAF-1 and NABP-1 (adapted from Gomez-Orte et al., 2019). **(B)** Immunofluorescence images of RAD-51 foci formed after ionizing radiation. *ints-6* knockdown impairs RAD-51 recruitment to DSBs following IR. As shown in the pictures, there are no RAD-51 foci before IR, either in worms fed the L4440 bacterial RNAi clone (panel 1, 5, 9) or fed the *ints-6* bacterial RNAi clone (panel 2, 6, 10). Following irradiation, RAD-51 foci can be observed in the gonads of worms treated with the L4440 bacterial RNAi clone (panel 3, 7, 11) but no RAD-51 foci are observed in the mitotic region of gonads of worms treated with the bacterial RNAi clone of *ints-6* (panel 4, 8, 12). Scale bar: 10 μm. Immunofluorescence images of phosphorylated CDK-1. *ints-6* knockdown abrogates Tyr15 CDK-1 phosphorylation in response to DNA damage. Tyr15 CDK-1 phosphorylation was detected in the nuclei of control gonads after irradiation (panel 15,19,23). However, in the gonads of worms knocked down for *ints-6* , phosphorylation of Tyr15 CDK-1 following IR was not detected (panel 16, 20, 24). Scale bar: 10 μm. **(C)** Proposed model. Upon the occurrence of a double strand break in DNA, the MRN complex together with ATM senses it. MRN then activates ATM triggering the DNA damage response pathway. One of the targets of ATM is hSSB1, which is phosphorylated and mobilized to the site of damage. It interacts directly with MRN complex stimulating its recruitment to the break site. The DNA end resection is then initiated, creating single stranded DNA overhangs. Based on our results and others previously published, we believe that at this point INTS-6 acts. These ssDNA ends are then coated with the RPA protein. Finally, RPA is replaced by RAD-51 and the resulting RAD-51 coated filament performs homology search and strand invasion, allowing DNA synthesis at the resected strand and subsequent repair.

## Description

The genome of all organisms is constantly being challenged by insults that result in DNA damage. Endogenous cellular metabolites, exogenous environmental hazards or replication errors may alter DNA sequence, structure or both. Although mutations can be beneficial on an evolutionary scale, accurate repair of DNA lesions is necessary to ensure genomic stability (Sperka et al., 2012; Marechal and Zou, 2013; Ta and Gioeli, 2014) . Among the different forms of DNA damage, double-strand breaks (DSBs) are the most toxic lesions because even a single DSB has the potential to activate cell cycle arrest, altering its growth and metabolism which can ultimately lead to cell death (Bennett et al., 1993) .

DSBs cause activation of ATM (ataxia-telangiectasia mutated) and ATR (ataxia-telangiectasia and Rad3-related) kinases, which are members of the phosphatidyl-inositol-3-OH kinase-like kinases (PIKK). Their activation leads to cell cycle arrest while the cell activates repair pathways and facilitates an open chromatin structure needed for repair (Marechal and Zou, 2013; Awasthi et al., 2015) .

To repair DNA DSBs and safeguard genome integrity, two main repair mechanisms are used in eukaryotes: homologous recombination (HR) and non-homologous end-joining (NHEJ) (Li and Heyer, 2008; Jasin and Rothstein, 2013) . The HR pathway, which is the primary focus of our study, is a high-fidelity repair route that uses an undamaged homologous DNA template from a sister chromatid or a homologous chromosome to provide the sequence information lost at the break site (Li and Heyer, 2008; Jasin and Rothstein, 2013; Schwertman et al., 2016) . In HR, after a DSB, a set of proteins promote DNA end resection producing 3' ssDNA overhangs that are rapidly coated by replication protein A (RPA). RPA is subsequently replaced by RAD51, which is one of the last proteins loaded onto the DSB. The resulting RAD51 coated filament performs homology search and strand invasion, allowing DNA synthesis at the resected strand and subsequent repair. The resulting joined molecule generated is processed by resolvases to terminate the repair process. (Schwertman et al., 2016) . If, in the end, DNA alterations cannot be repaired or tolerated, cells may enter senescence or even undergo apoptosis (Sperka et al., 2012; Marechal and Zou, 2013; Ta and Gioeli, 2014) .

One interesting aspect of the DNA damage response is the involvement of at least two mammalian Integrator complex subunits, Ints3 and Ints6 (Skaar et al., 2009; Zhang et al., 2009; Zhang et al., 2013) . Integrator is a complex of 15 subunits in mammals, that binds the C-terminal domain of RNA polymerase II to regulate snRNA 3'-end processing and gene expression (Baillat et al., 2005; Gardini et al., 2014; Offley et al., 2023) . In addition to its role in the complex, Ints3 interact with hSSB1 (human single stranded binding protein 1) and the uncharacterized protein C9orf80, forming a stable complex called the SOSS1 complex (Huang et al., 2009; Skaar et al., 2009; Zhang et al., 2013) . Although the exact mechanism by which it participates in the damage response is still unknown, there is considerable evidence indicating that Ints6 could also be part of this SOSS1 complex. Ints6 interaction is critical for maintaining hSSB1 protein level. Recently, the crystallographic structure of the complex has been solved, and it shows that Ints3 dimerize and interacts directly with Ints6 (Li et al., 2021; Jia et al., 2021) . Studies on hSSB1 demonstrated that after DNA damage, hSSB1 is phosphorylated by ATM kinase and localizes to DNA DSBs together with Nbs1, a member of the MRN complex along with Mre11 and Rad50. A recent study found that the recruitment or Nbs1 after DNA damage is promoted by SUMOylations of lysine residues K79 and K94 in the OB domain of hSSB1. Nbs1 also interacts whith the SOSS complex via Ints3 in a phospho-dependent manner. Hence, the recruitment of the MRN complex to the break site is accomplished by two parallel binding modes, one by binding of Nbs1 to hSSB1, and the other by binding of Nbs1 to Ints3, thought the exact order of recruitment is unknown. Meanwhile, the two others member of the MRN complex, Mre11 and Rad50, have also demonstrated to perform at least two important roles in resection: short-range endonucleolytic resection of 50 strands at DSB ends, and also stimulation of extensive resection through recruitment of 50 to 30 exonucleases and helicases **.** Although the exact mechanism remains unclear, hSSB1 is involved in recruitment of DNA repair proteins such as Rad51 and BRCA1 at DSBs. Therefore, cells deficient in hSSB1 display diminished capacity for DNA repair and enhanced genomic instability (Richard et al., 2008; Richard et al., 2011; Yang et al., 2013; El-Kamand et al., 2023) .

We previously immunoprecipitated *C. elegans* INTS-6 and detected its interacting protein partners by mass spectrometry (IP-MS). Apart from the rest of the members of the Integrator complex, we detected proteins involved in the DNA damage response, such as LAF-1 and NABP-1 , orthologs of the human DDX3X and hSSB, respectively (Gomez-Orte et al., 2019; Cargill et al., 2021; Randolph et al., 2024). Our data suggest that a possible SOSS complex composed of at least INTS-3 , INTS-6 and NABP-1 , is also present in *C. elegans* ( Fig. 1A ).

To verify the involvement of *C. elegans* INTS-6 in DNA repair by the HR pathway we checked for RAD-51 IRIF formation (irradiation induced foci) in response to X-radiation in the gonadal mitotic region of WT nematodes grown under standard conditions and upon *ints-6* knockdown. RAD-51 foci could be detected following irradiation in the gonadal mitotic region of worms fed with the empty vector control of bacterial RNAi clones. However, no RAD-51 IRIF were detected in the mitotic region of worm gonads depleted of *ints-6* ( Fig. 1B ). This result supports the idea that INTS-6 may be a key component of the *C. elegans* DNA damage response pathway and that it plays a role in RAD-51 recruitment to DSBs and DNA repair by the HR pathway. The fact that RNAi *ints-6* knockdown in *C. elegans* abrogates recruitment of RAD-51 to DSBs following IR suggests that INTS-6 acts upstream of RAD-51 in the HR DNA repair pathway. Studies in human cells demonstrated that depletion of Ints6, along with its paralog DDX26B, completely disrupted recruitment of Rad51 and reduced the accumulation of RPA IRIF (Zhang et al., 2013) .

To investigate the function of INTS-6 in the cell cycle arrest upon DNA damage, we performed immunostaining with pTyr15 Cdk-1 antibodies to show phosphorylated CDK-1 in the gonadal mitotic region after X-radiation. Cell cycle arrest by phosphorylation of CDK-1 is one of the most upstream events in the DNA damage response to allow repair proteins loading to DSBs (Parker and Piwnica-Worms, 1992; Booher et al., 1997; Liu et al., 1997) . Again, experiments were conducted in nematodes grown under standard conditions and upon *ints-6* knockdown. Tyr15 CDK-1 phosphorylation was not detected before irradiation either in the control or in the gonads knocked down for *ints-6* . As expected, following IR, phosphorylation of Tyr15 CDK-1 was clearly detected in the nuclei of control gonads. However, worms fed the bacterial RNAi clone of *ints-6* did not induce phosphorylation on Tyr15 CDK-1 ( Fig. 1B ). Strikingly, cells within the proliferation region of the gonads were able to arrest in response to IR although they did not phosphorylate Cdk1 Tyr15. This suggests that either RNAi depletion was not complete or, alternatively, redundant mechanisms may be acting to induce cell cycle arrest.

Cells must ensure the stability of genetic information during transcription. Several studies have linked transcription and DSB detection. Thus, members of the RNAP II-associated basal transcription machinery are involved in different DNA damage responses (Lainé and Egly, 2006; Derheimer et al., 2007) . It has been speculated that the potential presence of hSSB1, through its interaction with Ints3 and Ints6 at transcriptional pause sites might be key in maintaining genome integrity (Baillat and Wagner, 2015) . Our experiments indicate that *ints-6* plays a key role in the DNA damage response ( Fig. 1C ), not only recruiting repair proteins at the DSB sites but also controlling cell cycle progression in response to DNA damage.

## Methods

***C. elegans* culture on agar plates.** *C. elegans* strains were maintained as described by Brenner (1974) on Nematode Growth Medium (NGM) agar plates seeded with a lawn of *E.* *coli* OP50 , at 20ºC.

***C. elegans* synchronization (bleaching).** *C. elegans* eggs are surrounded by a shell that protects them from harmful environmental factors such as chemicals. This feature was used to obtain synchronized populations. Worms were harvested when the plates contained many gravid hermaphrodites and washed several times with M9 buffer. Worms were dissolved by treatment with bleaching solution (12% NaClO) for 10 min with vigorous shaking in between. To avoid an excess of the reaction and thereby damage to the embryos, destruction of the adult tissue was monitored under a dissecting microscope. The released eggs were collected by centrifugation (1500 rpm, 2 min) and then washed three times with M9 buffer to remove any hypochlorite residues. The eggs were left incubating in M9 buffer O/N (at 20ºC with rotation) to obtain a synchronized population since, in the absence of food, the hatched larvae arrested at the first larval stage (L1).

***C. elegans* RNA interference (RNAi).** Gene knockdown was carried out by RNA interference (RNAi), (Fire et al., 1998) , feeding the worms RNAi bacterial clones (Timmons and Fire, 1998) . *E.* *coli* HT115 was used to host the plasmid RNAi clones. In each RNAi experiment, an empty RNAi clone vector (L4440) was used as the control and compared to the *ints-6* RNAi clone.

5 ml LB medium containing ampicillin (100 μg/ml) was inoculated with a single bacterial colony and incubated at 37°C for 8 h with constant shaking. 400 μl of the bacterial culture was spread on 90 mm NGM RNAi feeding plates (NGM plates with 100 μg/ml ampicillin, 12.5 μg/ml tetracycline, 1 mM IPTG) and incubated O/N at RT to grow a bacterial lawn and induce dsRNA expression. The next day, synchronized L1 nematode populations were transferred to RNAi feeding plates. Experiments were performed at 20°C.

**X-radiation.** Worms were synchronized and the resulting L1 larvae were fed the bacterial RNAi clone of *ints-6* or the empty L4440 vector bacterial RNAi clone. Upon reaching the L4 larval stage, worms were irradiated in a CellRad machine (Precision) (90Gy). Twenty-four hours later, gonads were dissected, fixed and immunostained.

***C. elegans* germline isolation and immunostaining. *C. elegans* germline dissection, fixation and permeabilization.** First, 8 μl of dissection buffer (1X egg buffer, 0.02% Tween-20, 0.2 mM Levamisole and Milli-Q H20) (10X egg buffer: 1.18 M NaCl, 480 mM KCl, 20 mM CaCl2, 20 mM MgCl2, 250 mM HEPES pH 7.4) was placed in the center of a poly-L-lysine coated slide (Polysine® slides, Thermo Scientific). Young adult worms (8-10) were picked with a platinum wire and transferred to the dissection buffer. They were immediately dissected by cutting off either the head (just behind the pharynx) or the tail using the sharp tip of a needle. Gonads burst out and were carefully isolated from the rest of the worm. Next, 8 μl fixation buffer (1X egg buffer, 0.02% Tween-20, 4% formaldehyde, and Milli-Q H20) was added to the drop and mixed by pipetting 3 or 4 times. Then, 8 μl was removed from the mixture. Slides were incubated in Coplin jars filled with a pre-cooled (-20ºC) 1:1 acetone:methanol solution for 10 min. Next, slides were washed three times (10 min each) with 1% Triton PBS buffer followed by another 5 min wash with 0.1% Tween PBS.

***C. elegans* germ line blocking, antibody incubation and mounting.** When using Alexa dyes, slides were pre-blocked for 20-30 min using Image- iT® FX Signal Enhancer (Invitrogen). One drop was added and gently covered with a small piece of parafilm. Next, samples were blocked for 20-30 min in a Coplin jar with 10% fetal bovine serum diluted in 0.1% Tween PBS. Next, 35-45 μl of the corresponding primary antibody (see Reagents) was added to each slide and covered with a small piece of parafilm. Slides were incubated O/N at 4ºC in a humid chamber. The following day the primary antibody was washed three to four times (10 min each) with 0.1% Tween PBS in a Coplin jar and 35-45 μl of the corresponding secondary antibody (see Reagents) was added to each slide before they were again covered with a small piece of parafilm. Slides were incubated for 2 h in a dark box at RT. Next, the slides were washed three to four times (10 min each) with 0.1% Tween PBS in a Coplin jar in the dark. Finally, as much liquid as possible was wiped off and 8 μl of VECTASHIELD® Antifade Mounting Medium with DAPI were added into each cover slip (18 mm x 18 mm Zeiss Thickness no. 1 1⁄2 High performance). The cover slips were very gently placed over the slide with the worms. After a few minutes, slides were sealed with nail polish.

**Confocal microscopy.** Imaging was performed at the Department of Clinical Molecular Biology of Oslo University, with a Zeiss 780 confocal microscope. Images were acquired and processed using ZEN lite open software from Zeiss.

## Reagents

*C. elegans* strain used in this study:

N2 *C. elegans* WT isolate. Source: CGC ( *Caenorhabditis* Genetics Center, University of Minnesota, USA) https://cgc.umn.edu/

*E.coli* bacterial strains used in this study:

OP50 (Brenner, 1974) .

HT115 (Timmons et al., 2001; Ahringer, 2006) .

Plasmids used in this study:

L4440 (Timmons and Fire, 1998) .

*ints-6* (RNAi): (Rual et al., 2004) .

Antibodies used in this study:

**Table:** 

Primary Antibody	Dilution	Source
α-RAD-51	1:10000	SDIX 2948.00.02
α-pTyr15 CDK-1	1:1000	CALBIOCHEM 213940
Secondary Antibody	Dilution	Source
Goat Anti-Rabbit Alexa Fluor® 488	1:1000	Thermo Fisher Scientific

Media and buffers used to grow *C. elegans* :

Nematode Growth Medium (NGM) (Stiernagle, 2006) .

M9 Buffer (Stiernagle, 2006) .
